# Real-time imaging required for optimal echocardiographic assessment of aortic valve calcification

**DOI:** 10.1111/j.1475-097X.2012.01153.x

**Published:** 2012-07-29

**Authors:** Mohamed Yousry, Anette Rickenlund, Johan Petrini, Tomas Gustavsson, Ulrica Prahl, Jan Liska, Per Eriksson, Anders Franco-Cereceda, Maria J Eriksson, Kenneth Caidahl

**Affiliations:** 1 Department of Molecular Medicine and Surgery, Karolinska InstitutetStockholm, Sweden; 2 Department of Clinical Physiology, Karolinska University HospitalStockholm, Sweden; 3 Chalmers University of TechnologyGothenburg, Sweden; 4 Wallenberg Laboratory, Sahlgrenska Academy and University HospitalGothenburg, Sweden; 5 Department of Thoracic Surgery, Karolinska University HospitalStockholm, Sweden; 6 Atherosclerosis Research Unit, Center for Molecular Medicine, Department of Medicine, Karolinska InstitutetStockholm, Sweden

**Keywords:** aortic regurgitation, aortic stenosis, grey scale, sclerosis, ultrasound

## Abstract

**Introduction:**

Aortic valve calcification (AVC), even without haemodynamic significance, may be prognostically import as an expression of generalized atherosclerosis, but techniques for echocardiographic assessment are essentially unexplored.

**Methods:**

Two-dimensional (2D) echocardiographic recordings (Philips IE33) of the aortic valve in short-axis and long-axis views were performed in 185 consecutive patients within 1 week before surgery for aortic stenosis (*n* = 109, AS), aortic regurgitation (*n* = 61, AR), their combination (*n* = 8) or dilation of the ascending aorta (*n* = 7). The grey scale mean (GSMn) of the aortic valve in an end-diastolic short-axis still frame was measured. The same frame was scored visually 1–5 as indicating that the aortic valve was normal, thick, or had mild, moderate or severe calcification. The visual echodensity of each leaflet was determined real time applying the same 5-grade scoring system for each leaflet, and the average for the whole valve was calculated. Finally, a similar calcification score for the whole valve based on inspection and palpation by the surgeon was noted.

**Results:**

Visual assessment of real-time images using the proposed scoring system showed better correlation with the surgical evaluation of the degree of valve calcification (*r* = 0·83, *P*<0·001) compared to evaluation of stop frames by visual assessment (*r* = 0·66, *P*<0·001) or the GSMn score (*r* = 0·64, *P*<0·001). High inter- and intra-observer correlations were observed for real-time visual score (both intraclass correlation coefficient = 0·93).

**Conclusion:**

Real-time evaluation of the level of AVC is superior to using stop frames assessed either visually or by dedicated computer grey scale measurement software.

## Introduction

Aortic valve calcification/sclerosis (AVC) has been considered a natural degenerative consequence of ageing. However, its risk factors (including old age, male gender, hypertension, elevated lipoprotein levels, smoking and diabetes mellitus) and histopathological features (lipoprotein deposition, chronic inflammatory reaction and activation of calcification cascade) are similar to a generalized atherosclerosis process (Mohler *et al*., 1991; Lindroos *et al*., 1994; Otto *et al*., 1994; Stewart *et al*., 1997; Agmon *et al*., 2001). Although AVC is often associated with aortic stenosis (AS), it may exist without stenosis and is then echocardiographically defined as focal areas of increased echogenicity and thickening of aortic valve leaflets without restriction of leaflet motion (Otto, 2004). The prevalence of AVC without significant AS is 20–30% in patients over 65 years of age (Stewart *et al*., 1997; Otto *et al*., 1999), reaching 48–57% at the age of 80 (Lindroos *et al*., 1994; Stewart *et al*., 1997). The degree of AVC predicts the progression of asymptomatic AS (Rosenhek *et al*., 2004). Furthermore, AVC has been found to be a strong predictor of cardiovascular morbidity and mortality in the general population, with approximately 50% increase in the risk of cardiovascular death and myocardial infarction (Otto *et al*., 1999). AVC may also predict coronary artery disease (CAD) to a greater extent than gender, hypertension, family history and hypercholesterolaemia (Acarturk *et al*., 2003) and has been demonstrated to be associated with a higher prevalence of left ventricular (LV) hypertrophy, ventricular arrhythmias, myocardial infarction and systolic heart failure (Palmiero *et al*., 2007). The calcific degenerative process that is present in mild and moderate AS is associated with increased mortality (Otto *et al*., 1999; Wilson *et al*., 2001; Rosenhek *et al*., 2004). With the increasing awareness of the predictive power of AVC, reliable non-invasive methods allowing quantitative assessment of its severity are needed.

Echocardiography is the most commonly used imaging technique for the assessment of AVC. However, current visual assessment of calcifications from the ultrasonic images is subjective, largely depending on the expertise of the examiner and vulnerable to inter- and intra-observer variability, especially when a well-defined and validated scoring system is lacking (Poggianti *et al*., 2003; Messika-Zeitoun *et al*., 2004; Palmiero *et al*., 2007; Zapolski *et al*., 2008).

Quantitative analysis of vascular plaque morphology and content using a dedicated grey scale measurement software has shown promising results (Griffin *et al*., 2007; Prahl *et al*., 2010). In AVC evaluation, limited initial attempts have been made using acoustic densitometry (Sgorbini *et al*., 2004).

The present echocardiographic study aimed at establishing an AVC scoring system to improve reproducibility and reliability of the echocardiographic assessment of AVC. The study was also designed to compare visual assessment and computer-based grey scale determination of AVC by ultrasound, to scoring by inspection and palpation at valve replacement.

## Methods

One hundred eighty-five patients (57 women) participated in this study. All patients were accepted for aortic valve and/or aortic root surgery within a prospective single centre study (Advanced Study of Aortic Pathology), which is performed at the Karolinska University Hospital, Stockholm, Sweden. AS was the dominant aortic valve lesion in 109 (59%) patients, aortic regurgitation (AR) in 61 (33%), combined AS and AR in 8 (4%), and ascending aorta aneurysm without significant valvular pathology in 7 (4%) patients. Significant CAD, defined as >50% lesion in any large or medium size coronary artery, was excluded by diagnostic coronary angiography in all patients.

The local ethics committee approved the study protocol, and all patients gave their informed consent to participate.

All patients underwent comprehensive transthoracic echocardiography (TTE) using a Philips IE33 ultrasound scanner with S5-1 transducer (1–5 MHz) (Philips Healthcare, Best, the Netherlands). Aortic valve morphology and function were assessed by an experienced echocardiographer. Studies were performed within 1 week of surgery, and all images were stored digitally for offline analysis. We did not exclude any patient because of poor image quality.

Computer software developed for atherosclerotic plaque analysis (Artery Measurement System, AMS) in collaboration between the Physiology Group at the Wallenberg Laboratory (http://www.wlab.gu.se), Gothenburg University and Department of Signals and Systems at Chalmers University of Technology, Gothenburg, Sweden) was used for grey scale measurements of the aortic valve leaflets (Wendelhag *et al*., 1997). A parasternal short-axis still frame with the aortic valve leaflets fully closed at end diastole was used. For each patient, a grey scale calibration was performed using a sample of the intravascular blood pool (avoiding areas of noise) as the black reference (score 0) and the brightest part of the pericardium or a calcium spot within the leaflets, if the pericardium was not visualized in the image used, as the white reference (score value 255) (Griffin *et al*., 2007). A region of interest was manually drawn around the aortic valve leaflets, excluding the annulus, and grey scale mean (GSMn) of the valve was then automatically calculated by the AMS program. We used GSMn rather than median as valvular calcification may be unevenly distributed.

The same short-axis still frame as used by the grey scale software, with the aortic valve leaflets fully closed at end diastole, was also assessed visually by one observer. The 5-degree scoring system is summarized in Table 1. Calcification was defined as bright echogenic spots within a leaflet, while sclerosis was defined as thickened leaflets with no bright echogenic spots. A single score value from 1 to 5 according to Table 1 was assigned to the whole valve (still frame visual score, SVS).

**Table 1:** Aortic valve calcification scoring system

Grade	Description
1	Normal leaflets with no evidence of thickening or calcification
2	Evidence of thickening (sclerosis) but no evidence of calcification
3	Calcification (small calcium spot(s) not exceeding one-third of the leaflet area)
4	Moderate calcification (calcium not exceeding two-thirds of the leaflet area)
5	Heavily calcified (calcification covering more than two-thirds of the leaflet area)

In real-time visual score (RVS), each leaflet was given a single score, and the mean for the valve was calculated.

In intra-operative score (IOS) and still frame visual score (SVS), the whole valve was given a single score.

Digitally stored 2D real-time short-axis images of the aortic valve leaflets were also assessed visually. Every single aortic valve leaflet was given a score, and an average score was calculated whether tricuspid or bicuspid valve (real-time visual score, RVS). The procedure was repeated by another independent investigator.

**Figure 1:**
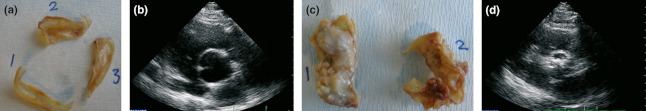
Valves from the study: tricuspid mildly thickened valve (a, b) and bicuspid severely calcified valve (c, d).

Two surgeons operated on all patients in the study and described the degree of calcification of the aortic valve leaflets. The investigator could scrutinize the appearance of the valve *in situ* and after explantation and explore the roughness, thickness and weight of the valve by palpation. A single intra-operative score (IOS) for the whole valve was assigned following the same scoring system as in the echocardiographic assessment (Table 1). The IOS was used as the standard for comparison of visual scoring of TTE images as well as GSM of the aortic valve. Some examples of the valves from our study are shown in Fig. 1.

### Statistical analysis

All statistical calculations were done using commercially available statistics software PASW statistics 17.0.2 by SPSS/IBM, Chicago, IL, USA. Descriptive statistics were presented as mean and standard deviation if normal distributed. Comparisons and correlations between different AVC assessment methods were done using Student's *t*-test, Pearson's and Spearman's rank correlation coefficient (*r*) as applicable. We used Wilcoxon's test for paired samples and Mann–Whitney's *U*-test for unpaired samples. *P*<0·05 was regarded as significant. Inter- and intra-observer variability were analyzed using intraclass correlation coefficient (ICC), with values above 0·75 representing good correlation and values between 0·4 and 0·75 fair reproducibility.

## Results

Within the study group, 104 patients had bicuspid aortic valve, representing 56% of the study population (six of these patients were surgically classified as having unicuspid valves). Baseline characteristics and echocardiographic measurements of the 185 patients studied are summarized in Tables 2 and 3. Patients with predominant AS (*n* = 109) tended to be older, with a median age of 69 years and including more women (39%) compared to patients with predominant AR (*n* = 61) with a median age of 58 years and 18% women.

**Table 2:** Patients characteristics

		Bicuspid group (*n* = 104)		Tricuspid group (*n* = 81)	
			
	All patients (*n* = 185)	<65 years (*n* = 66)	≥65 years (*n* = 38)	<65 years (*n* = 25)	≥65 years (*n* = 56)
AS *n* (%)	109 (59)	34 (52)	34 (89)	4 (16)	37 (66)
AR *n* (%)	61 (33)	24 (36)	1 (3)	20 (80)	16 (28)
AS/AR *n* (%)	8 (4)	5 (8)	2 (5)	0	1 (2)
AAD *n* (%)	7 (4)	3 (5)	1 (3)	1 (4)	2 (4)
Females *n* (%)	57/185 (31)	15/6 (23)	14/38 (37)	4/25 (16)	24/56 (43)
Weight (kg)	82 ± 15	85 ± 14	77 ± 12	88 ± 11	80 ± 17
Height (cm)	175 ± 10	178 ± 9	173 ± 8	178 ± 9	171 ± 11
BSA (m^2^)	1·97 ± 0·2	2·03 ± 0·18	1·9 ± 0·17	2·06 ± 0·15	1·92 ± 0·23
Mean age (years)	63·9 ± 11·6	54·6 ± 8·9	72·1 ± 4·8	54·4 ± 8·2	73·6 ± 5·1

AAD, aortic aneurysm or dissection; AS, aortic stenosis; AR, aortic regurgitation; BSA, body surface area.

**Table 3:** Echocardiographic data

	Bicuspid valve (*n* = 93)		Tricuspid valve (*n* = 77)	
		
Variables	AS (*n* = 68)	AR (*n* = 25)	AS (*n* = 41)	AR (*n* = 36)
Aortic root, ED diameter (mm)	36·1 ± 5·8	42·0 ± 6·7	32·1 ± 4·3	42 ± 6·9
Ascending aorta, ED diameter (mm)	40·4 ± 7·6	44·6 ± 7·6	33·8 ± 4·6	43·4 ± 9·4
Ascending aorta, ED diameter > 40 mm (*n*)	29 (43%)	18 (72%)	3 (7%)	18 (50%)
LVOT, ES diameter (mm)	22·8 ± 2·8	27·4 ± 3·9	20·8 ± 1·8	24·4 ± 3·4
AV area (cm^2^)	0·9 ± 0·4	3·0 ± 1·0	0·8 ± 0·3	3·2 ± 1·1
AV peak gradient (mmHg)	80·1 ± 31·4	20·4 ± 14·4	77·6 ± 19·9	15·4 ± 8·0
AV mean gradient mean (mmHg)	51·1 ± 19·7	12·0 ± 8·7	48·9 ± 13·5	8·0 ± 3·9
Surgical AVC score (score 1-5)	4·5 ± 0·7	2·5 ± 1·1	4·3 ± 0·9	1·4 ± 0·6
Grey scale mean (grey level 0-255)	78·0 ± 18·2	44·2 ± 18·8	91·3 ± 22·1	34·8 ± 13·5

AR, aortic regurgitation; AS, aortic stenosis; AV, aortic valve; ED, end diastolic, ES, end systolic, AVC, aortic valve calcification; LVOT, left ventricular outflow tract.

Patients with bicuspid valves were younger with a median age of 62 years versus 70 years in the tricuspid group. The median age of the whole study population was 65 years, and lesions were categorized into two age groups, group I <65 years and group II ≥ 65 years. Baseline characteristics for these groups are shown in Table 2. Patients with bicuspid valves had more often AS (*n* = 68, 65%) than AR (*n* = 25, 24%). AR as dominating lesion in the bicuspid valve group was essentially limited to those below 65 years of age (*n* = 24/25, 96%). The tricuspid valve group showed a more equal representation of AS (*n* = 41) and AR (*n* = 36). With tricuspid valves, in contrast to the bicuspid valve group, AR was more evenly distributed below and above the whole group′s median age (65 years) and AS demanding surgery was found mainly after the age of 65 (*n* = 37/41, 90%). Combined AS/AR was observed less frequently in tricuspid valve (*n* = 1, 1·2%) than in the bicuspid valve group (*n* = 7, 6·7%) with a majority of the lesions requiring surgical intervention before 65 years of age. A higher percentage of dilated aortic root (52%) was observed in the bicuspid valve group, reaching up to 72% in bicuspid patients with AR, in comparison with only 30% in the tricuspid valve group and as low as 7% in tricuspid valves with AS.

A summary of the correlations between different techniques of AVC assessment and IOS as a reference, and between visual and GSM assessment of still frames, is shown in Table 4. Visual real-time assessment showed a good correlation with IOS for the two investigators, and this was confirmed when the subgroups of different valve lesions and valve types were assessed. On the other hand, visual interpretation of still frames showed weaker correlations with the IOS, not improved by GSMn. Similar results were noted regarding correlations between the two still frame-based methods (GSM and SVS) and IOS, and these results were unchanged when considering the subgroups with AS/AR and bi/tricuspid valves.

**Table 4:** Correlations between studied variables

	Inter observer RVS^1,2^	Intra-observer RVS^1,1^	RVS^1^ versus GSMn	RVS^2^ versus GSMn	RVS^1^ versus IOS	RVS^2^ versus IOS	GSMn^1^ versus IOS	SVS^1^ versus IOS
Bicuspid	*r* = 0·88	*r* = 0·89	*r* = 0·74	*r* = 0·72	*r* = 0·78	*r* = 0·76	*r* = 0·56	*r* = 0·57
Tricuspid	*r* = 0·95	*r* = 0·96	*r* = 0·93	*r* = 0·89	*r* = 0·83	*r* = 0·84	*r* = 0·73	*r* = 0·72
All	*r* = 0·93	*r* = 0·94	*r* = 0·84	*r* = 0·82	*r* = 0·83	*r* = 0·82	*r* = 0·64	*r* = 0·66

All correlations were significant with *P*<0·001.

GSMn, grey scale mean; IOS, intra-operative score; RVS, real-time images visual score; SVS, still frame visual score.

Index numbers mean observer 1 and observer 2.

Observer variability in terms of inter- and intraclass correlation (ICC) coefficients was for the real-time scoring 0·93 and 0·93, and for still frame scoring 0·90 and 0·85.

## Discussion

To our knowledge, this study is the first to evaluate computer-based versus visual assessment of AVC from echocardiographic images in comparison with intra-operative surgical assessment. The intra-operative AVC estimation made by the surgeon was regarded as the gold standard in this study. We demonstrated that application of cine loops was possible with a high inter- and intra-observer reproducibility and improved the correlation to surgical findings, in comparison with the use of only diastolic still images. While the grey scale evaluation is less subjective, it was not closer related to the surgical score than the visual assessment of corresponding still frames.

Grey scale measurement software has been successfully used for characterization of plaques in the carotid arteries (Gronholdt *et al*., 1998; Griffin *et al*., 2007; Prahl *et al*., 2010). We used GSMn in contrast to grey scale median commonly used for plaque characterization as, in contrast to median, it is affected by small calcifications giving high echo reflection. There may be several reasons why, despite GSMn is a more continuous and potentially less investigator-dependent measure than the visual score, it did not significantly increase the agreement with the IOS compared with that of SVS. Even with the evaluation of carotid plaque, the major advantage of GSM is the user independence and reproducibility, rather than a superiority in classification. As a matter of fact, the human eye has an excellent ability of pattern recognition used as a basis and reference also for recent development of grey scale algorithms (Prahl *et al*., 2010). Further, GSMn has in common with the visual interpretation the fact that the evaluation of a still frame does not necessarily mirror the average or full extent of calcification.

With a single stop frame, the visualization of calcified parts is dependent on an optimal transection of the leaflets. As the calcification process can cause thick irregular chunks as well as thin calcified slices, and also calcification at different depth of the valve thickness, it is logical that a short-axis ultrasonic still frame cannot represent a complete evaluation of the disease process. Thus, in a single image, small calcifications may be outside of the imaged plane while clearly visible at some point in time during a heart cycle. It was therefore not surprising that a better correlation to the IOS was obtained by visual evaluation of AVC from the moving valve in real-time imaging, allowing visualization of all parts of the valve passing the interrogation plane during a full heart cycle. In the current study, we used the parasternal short-axis view where all leaflets can be interpreted. Such approach is common (Nucifora *et al*., 2009; Corciu *et al*., 2010). Although a number of studies have applied echocardiographic scoring of AVC, the results have mainly been used to evaluate prognostic power (Otto *et al*., 1999; Rosenhek *et al*., 2000), and the possibility to predict, for example, coronary disease (Nucifora *et al*., 2009; Corciu *et al*., 2010; Pressman *et al*., 2011) or AR after transcatheter aortic valve implantation (Colli *et al*., 2011). Information on the accuracy of echocardiographic evaluation of AVC is scarce. Thus, few comparisons exist between echocardiographic determination of valvular calcification and other techniques, although total cardiac calcification scoring by ultrasound compares favourably to computer tomography (Corciu *et al*., 2010; Colli *et al*., 2011). In the scoring system developed by Corciu *et al*., the leaflets were scored separately as normal (=0), with enhanced echogenicity (=1) or calcified (=2) yielding a total maximum of six for a tricuspid valve. This score was developed from the one suggested by Tolstrup *et al*. (2002), which contained 5 degrees, no thickening (=0), reflectance slightly (=1), moderately (=2) increased or generalized (=3), or marked with thickness >6 mm (=4), while the other degrees had less but specified thickening. With this scale, grades 0–1 were regarded normal, and 2–4 mildly, moderately or severely sclerotic. The highest score given to one of the cusps was regarded representative for the degree of sclerosis of the valve. These scales were based on the evaluation of short-axis images and so was also the somewhat more simple score by Nucifora *et al*. (2009) where aortic valve sclerosis was denoted absent (=0), mild (=1), moderate (=2) or severe (=3). As outlined in Table 1, our scale was rather similar, with the wording ‘sclerosis’ exchanged for ‘calcification’. Moreover, we added ‘thickened’, presupposing that an early stage of thickening would precede calcification and be recognizable by the surgeon as well as on echocardiography. This presumption and the feasibility of our scale were shown valid in the present study, and the five steps turned out to be intuitive and easy to apply for echocardiographers and surgeons. Our reproducibility with ultrasound scoring was very good and comparable to studies using computed tomography and the Agatston score to assess AVC (Koos *et al*., 2004; Messika-Zeitoun *et al*., 2004; Budoff *et al*., 2006).

Although the prevalence of bicuspid aortic valves in the general population is only 1–2%, bicuspid valves are much more common, around 20–50%, in patients subjected to aortic valve surgery (Turina *et al*., 1986; Bauer *et al*., 2007; Jackson *et al*., 2011). Interestingly, our study showed better correlation to IOS for tricuspid compared to bicuspid valves regarding AVC, particularly regarding interpretation of still frames by visual inspection and GSMn evaluation. The explanation for this is unclear, but a more complex and diverted anatomy of the bicuspid valve might be less favourable for assessment. Further, the deviant three-dimensional anatomy of the bicuspid valve with an echo dense raphe could contribute to make the choice of a single representative short-axis image more difficult.

There are some particular strengths and some limitations with this study. Our gold standard, the IOS, allows the investigator to both scrutinize the appearance and explore the roughness and thickness of the valve by palpation, but it is semi-quantitative and partly subjective. The use of a similar system for IOS and ultrasound interpretation is an advantage. Comparison to an absolute measurement of calcium content is lacking. Varying ultrasound transmission and sometimes poor image quality are limitations in evaluating TTE images. However, we did not exclude patients for poor image quality to make the comparisons as clinically relevant as possible.

In conclusion, we find GSMn evaluation as accurate as visual grading to determine degree of AVC, and for interpretation of still frames, its less subjective nature is an advantage if the goal is to, for example, compare longitudinal alteration in calcification over time. However, visual evaluation of real-time images seems to be closer related to intra-operative assessment than either GSMn or visual interpretation of still frames. Software that is capable of assessing a full real-time loop during the specific time when the valve is visible may improve the estimation of AVC in a more quantitative way, while also being more cumbersome. The applied 5-grade scoring system for assessing AVC is feasible and reproducible and can be successfully used to reduce subjectivity in assessing AVC.

## References

[b1] Acarturk E, Bozkurt A, Cayli M, Demir M (2003). Mitral annular calcification and aortic valve calcification may help in predicting significant coronary artery disease. Angiology.

[b2] Agmon Y, Khandheria BK, Meissner I, Sicks JR, O'Fallon WM, Wiebers DO, Whisnant JP, Seward JB, Tajik AJ (2001). Aortic valve sclerosis and aortic atherosclerosis: different manifestations of the same disease? Insights from a population-based study. J Am Coll Cardiol.

[b3] Bauer M, Bauer U, Siniawski H, Hetzer R (2007). Differences in clinical manifestations in patients with bicuspid and tricuspid aortic valves undergoing surgery of the aortic valve and/or ascending aorta. Thorac Cardiovasc Surg.

[b4] Budoff MJ, Takasu J, Katz R, Mao S, Shavelle DM, O'Brien KD, Blumenthal RS, Carr JJ, Kronmal R (2006). Reproducibility of ct measurements of aortic valve calcification, mitral annulus calcification, and aortic wall calcification in the multi-ethnic study of atherosclerosis. Acad Radiol.

[b5] Colli A, D'Amico R, Kempfert J, Borger MA, Mohr FW, Walther T (2011). Transesophageal echocardiographic scoring for transcatheter aortic valve implantation: impact of aortic cusp calcification on postoperative aortic regurgitation. J Thorac Cardiovasc Surg.

[b6] Corciu AI, Siciliano V, Poggianti E, Petersen C, Venneri L, Picano E (2010). Cardiac calcification by transthoracic echocardiography in patients with known or suspected coronary artery disease. Int J Cardiol.

[b7] Griffin M, Nicolaides A, Kyriacou E (2007). Normalisation of ultrasonic images of atherosclerotic plaques and reproducibility of grey scale median using dedicated software. Int Angiol.

[b8] Gronholdt ML, Nordestgaard BG, Wiebe BM, Wilhjelm JE, Sillesen H (1998). Echo-lucency of computerized ultrasound images of carotid atherosclerotic plaques are associated with increased levels of triglyceride-rich lipoproteins as well as increased plaque lipid content. Circulation.

[b9] Jackson V, Petrini J, Caidahl K, Eriksson MJ, Liska J, Eriksson P, Franco-Cereceda A (2011). Bicuspid aortic valve leaflet morphology in relation to aortic root morphology: a study of 300 patients undergoing open-heart surgery. Eur J Cardiothorac Surg.

[b10] Koos R, Mahnken AH, Sinha AM, Wildberger JE, Hoffmann R, Kuhl HP (2004). Aortic valve calcification as a marker for aortic stenosis severity: assessment on 16-mdct. AJR Am J Roentgenol.

[b11] Lindroos M, Kupari M, Valvanne J, Strandberg T, Heikkila J, Tilvis R (1994). Factors associated with calcific aortic valve degeneration in the elderly. Eur Heart J.

[b12] Messika-Zeitoun D, Aubry MC, Detaint D, Bielak LF, Peyser PA, Sheedy PF, Turner ST, Breen JF, Scott C, Tajik AJ, Enriquez-Sarano M (2004). Evaluation and clinical implications of aortic valve calcification measured by electron-beam computed tomography. Circulation.

[b13] Mohler ER, Sheridan MJ, Nichols R, Harvey WP, Waller BF (1991). Development and progression of aortic valve stenosis: atherosclerosis risk factors–a causal relationship? A clinical morphologic study. Clin Cardiol.

[b14] Nucifora G, Schuijf JD, van Werkhoven JM, Jukema JW, Marsan NA, Holman ER, van der Wall EE, Bax JJ (2009). Usefulness of echocardiographic assessment of cardiac and ascending aorta calcific deposits to predict coronary artery calcium and presence and severity of obstructive coronary artery disease. Am J Cardiol.

[b15] Otto CM (2004). Why is aortic sclerosis associated with adverse clinical outcomes?. J Am Coll Cardiol.

[b16] Otto CM, Kuusisto J, Reichenbach DD, Gown AM, O'Brien KD (1994). Characterization of the early lesion of ‘degenerative’ valvular aortic stenosis. Histological and immunohistochemical studies. Circulation.

[b17] Otto CM, Lind BK, Kitzman DW, Gersh BJ, Siscovick DS (1999). Association of aortic-valve sclerosis with cardiovascular mortality and morbidity in the elderly. N Engl J Med.

[b18] Palmiero P, Maiello M, Passantino A, Wasson S, Reddy HK (2007). Aortic valve sclerosis: is it a cardiovascular risk factor or a cardiac disease marker?. Echocardiography.

[b19] Poggianti E, Venneri L, Chubuchny V, Jambrik Z, Baroncini LA, Picano E (2003). Aortic valve sclerosis is associated with systemic endothelial dysfunction. J Am Coll Cardiol.

[b20] Prahl U, Holdfeldt P, Bergstrom G, Fagerberg B, Hulthe J, Gustavsson T (2010). Percentage white: a new feature for ultrasound classification of plaque echogenicity in carotid artery atherosclerosis. Ultrasound Med Biol.

[b21] Pressman GS, Crudu V, Parameswaran-Chandrika A, Romero-Corral A, Purushottam B, Figueredo VM (2011). Can total cardiac calcium predict the coronary calcium score?. Int J Cardiol.

[b22] Rosenhek R, Binder T, Porenta G, Lang I, Christ G, Schemper M, Maurer G, Baumgartner H (2000). Predictors of outcome in severe, asymptomatic aortic stenosis. N Engl J Med.

[b23] Rosenhek R, Klaar U, Schemper M, Scholten C, Heger M, Gabriel H, Binder T, Maurer G, Baumgartner H (2004). Mild and moderate aortic stenosis. Natural history and risk stratification by echocardiography. Eur Heart J.

[b24] Sgorbini L, Scuteri A, Leggio M, Leggio F (2004). Association of mitral annulus calcification, aortic valve calcification with carotid intima media thickness. Cardiovasc Ultrasound.

[b25] Stewart BF, Siscovick D, Lind BK, Gardin JM, Gottdiener JS, Smith VE, Kitzman DW, Otto CM (1997). Clinical factors associated with calcific aortic valve disease. Cardiovascular health study. J Am Coll Cardiol.

[b26] Tolstrup K, Roldan CA, Qualls CR, Crawford MH (2002). Aortic valve sclerosis, mitral annular calcium, and aortic root sclerosis as markers of atherosclerosis in men. Am J Cardiol.

[b27] Turina J, Turina M, Krayenbuhl HP (1986). Significance of the bicuspid aortic valve in the incidence of aortic valve defects in adults. Schweiz Med Wochenschr.

[b28] Wendelhag I, Liang Q, Gustavsson T, Wikstrand J (1997). A new automated computerized analyzing system simplifies readings and reduces the variability in ultrasound measurement of intima-media thickness. Stroke.

[b29] Wilson PW, Kauppila LI, O'Donnell CJ, Kiel DP, Hannan M, Polak JM, Cupples LA (2001). Abdominal aortic calcific deposits are an important predictor of vascular morbidity and mortality. Circulation.

[b30] Zapolski T, Wysokinski A, Janicka L, Grzebalska A, Ksiazek A (2008). Aortic stiffness and valvular calcifications in patients with end-stage renal disease. Pol Arch Med Wewn.

